# Comparison of angle, shape, and position of articular processes in Dobermans and Great Danes with and without cervical spondylomyelopathy

**DOI:** 10.1186/s12917-017-0997-4

**Published:** 2017-03-24

**Authors:** Marília de Albuquerque Bonelli, Ronaldo Casimiro da Costa, Paula Martin-Vaquero, Carolina Gonçalves Dias Lima

**Affiliations:** 1 Department of Veterinary Clinical Sciences, College of Veterinary Medicine, The Ohio State University, 601 Vernon Tharp St., Columbus, OH 43210 USA; 2 Department of Veterinary Medicine, Federal Rural University of Pernambuco, R. Dom Manoel de Medeiros s/n, Recife, PE 52171-900 Brazil; 3 Ogilvy Healthworld, Avda. de Burgos 21, C. P. 28036 Madrid, Spain

**Keywords:** Articular facets, Cervical spine, Facet joint, Wobbler syndrome, Zygapophyseal joint

## Abstract

**Background:**

Cervical spondylomyelopathy (CSM), also known as wobbler syndrome, affects mainly large and giant-breed dogs, causing compression of the cervical spinal cord and/or nerve roots. Structural and dynamic components seem to play a role in the development of CSM; however, pathogenesis is not yet fully understood. Physiologic and pathologic movements of the cervical spine depend on the morphology and morphometry of articular processes, as well as on intervertebral discs and vertebral column ligaments. Moreover, the characteristics of the articular processes affect motion and stability of the vertebral column. The goal of this study was to investigate the angle, shape, and position of the articular surfaces within the articular processes and compare them between Doberman Pinschers and Great Danes with and without cervical spondylomyelopathy.

**Results:**

Magnetic resonance images were obtained for 60 dogs: 15 clinically normal Dobermans (Dob-N), 15 CSM-affected Dobermans (Dob-CSM), 15 clinically normal Great Danes (GD-N), and 15 CSM-affected Great Danes (GD-CSM). Angle, shape, and position (lateral distance) of the articular surfaces from the articular processes were analyzed from C_2–3_ to C_7_-T_1_. Results indicate that the mean angle was different between Dob-CSM and GD-CSM at C_4–5_, C_5–6_, and C_6–7_, and between GD-N and GD-CSM at C_6–7_. There were differences between Dob-N and GD-N, and between Dob-CSM and GD-CSM for the lateral distance at most locations, except C_2–3_. Compared with Great Danes, Dobermans generally had a greater proportion of concave caudal surfaces at C_4–5_, C_5–6_, and C_6–7_. Concave articular surfaces have been associated with greater axial rotation. This may explain the high proportion of disc-associated CSM in Dobermans compared to Great Danes. The differences between breeds suggest they may have different motion patterns in the caudal cervical vertebral column.

**Conclusions:**

Considering that no differences in angle, shape, or position of the articular surfaces within the articular processes were found between normal and CSM-affected dogs, their relevance appears to have a secondary role in the pathogenesis of CSM.

**Electronic supplementary material:**

The online version of this article (doi:10.1186/s12917-017-0997-4) contains supplementary material, which is available to authorized users.

## Background

Cervical spondylomyelopathy (CSM), commonly known as wobbler syndrome, is a disorder characterized by compression of the cervical spinal cord and/or nerve roots [1, 2] and can be divided into osseous-associated (OA-CSM) and disc-associated (DA-CSM), although overlap exists [1]. Structural changes of the cervical vertebral column, along with dynamic components, appear to be involved in the pathogenesis of CSM; however, the individual contribution of each is not yet fully understood [1, 2]. Dynamic lesions, where compression worsens as the amount of the space available within the vertebral canal increases or decreases depending on flexion and extension of the neck [1], are thought to be in part a consequence of the decrease in vertebral canal diameter during cervical extension [3].

Physiologic and pathologic movements of the cervical spine depend on the articular process joints which, along with the intervertebral discs and the vertebral column ligaments, facilitate the transfer of loads and guide and constrain the motion of the vertebrae [4]. Morphology, morphometry, and angle of the articular surfaces of the articular processes could affect the mechanical properties and patterns of motion of the articular process joints. As such, variations in articular process joint characteristics between breeds or vertebral levels could thus result in different motion patterns and be relevant to the pathogenesis of CSM [4–7]. A smaller horizontal angle, curved articular surfaces, and shorter distance from the center of the articular joint to the dorsal rim of the intervertebral disc have been associated with greater mobility [6, 7].

The objective of the present study was to investigate whether there are differences in the angle, shape, and position of the articular surfaces that form the articular processes between Doberman Pinscher and Great Dane dogs with and without CSM. Our primary hypothesis was that the articular surfaces would have a lower horizontal inclination angle, be more ventrally positioned, and with curved caudal articular surfaces in dogs with CSM. A second hypothesis was that there would be a significant difference between Dobermans and Great Danes, with Dobermans being supposed as having the aforementioned characteristics that would be considered related with greater mobility.

## Methods

Magnetic resonance (MR) images of the cervical vertebral column of Doberman Pinscher and Great Dane dogs were retrospectively reviewed. All dogs had undergone thorough physical and neurological examinations. The MR studies consisted of at least sagittal and transverse T1- and T2-weighted images from C_2_ to T_1_, acquired using high-field magnets (1.5 T Magnetom Vision, Siemens; 3.0 T Achieva, Philips Healthcare), with a minimum of three transverse slices at each intervertebral disc level. The transverse images had been obtained parallel to the vertebral endplate. Slice thickness was 3 mm, with no interslice interval. For imaging, all dogs had received premedication with an opioid and a benzodiazepine or acepromazine and had been under general anesthesia using propofol and isoflurane and positioned in dorsal recumbency, with their cervical vertebral columns centered, extended and held in place with sandbags.

Dogs were included in the CSM-affected group if they had a confirmed diagnosis of CSM based on history, clinical signs, and presence of compatible magnetic resonance imaging (MRI) changes. Dogs were considered clinically normal based on absence of neurologic abnormalities at the time of the MRI scan and no history of neurologic disease. Dogs would have been excluded if they had unclear images of the articular surfaces, or MR image artifacts that interfered with visualization. All dogs were part of prospective studies carried out by the senior author [8–10].

T1-weighted images were used to obtain the measurements and perform the morphological analysis because they allowed clearer delimitation of the articular processes and their articular surfaces. All regions, from C_2–3_ to C_7_-T_1,_ were studied. All analyses were performed by the same investigator (MAB), who was blinded to the clinical presentation of the dogs.

**Fig. 1:**
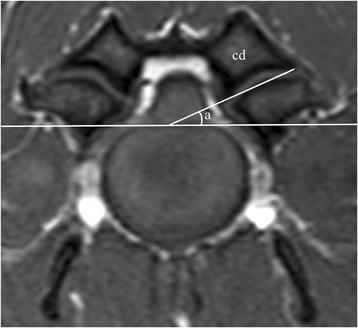
Transverse T1-weighted magnetic resonance image from a CSM-affected Great Dane, demonstrating the measurement of the facet angle (**a**) of the *left* caudal articular process (cd)

**Fig. 2:**
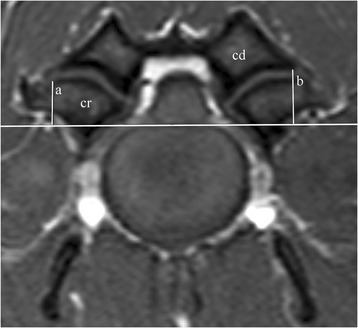
Transverse T1-weighted magnetic resonance image from a clinically normal Doberman Pinscher, demonstrating the cranial lateral (**a**) and caudal lateral (**b**) distance points for the cranial (cr) and caudal (cd) articular processes

For measurements regarding the angle (Fig. 1) and lateral distance (Fig. 2) of the articular surfaces of the articular processes, the image with the clearest view of the articular surfaces for each articular process joint was selected. This was regularly the image that represented the middle of the articular joint. When two images were alike, the one closest to the cranial endplate of the caudal vertebra was selected. First, a vertical line was then drawn bisecting the spinous process and the intervertebral disc/vertebral body. Then, another line was drawn perpendicular to the first, with the most dorsal point of the floor of the vertebral canal as the reference line (Fig. 1). The articular surface of an articular process was considered to extend medially or laterally until a change in hyperintensity of the articular surface was observed. Both T1- and T2-weighted images were used to distinguish the articular surface from the hyperintense fat surrounding the articular process joint. All data were collected separately for left and right cranial and caudal articular surfaces/processes.

### Angle of the articular surfaces

A line was drawn from the most lateral to the most medial point of the articular surface of each articular process, regardless of shape variations between these points. The angle was then determined in relation to the horizontal reference line (Fig. 1). This method was adapted from previous studies [6, 11, 12].

### Shape of the articular surfaces

**Fig. 3:**
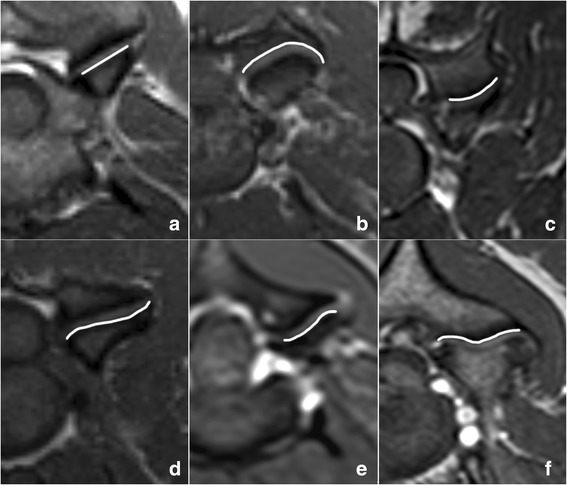
Transverse T1-weighted magnetic resonance images illustrating the classification of the articular surface shapes: plane articular surface in a CSM-affected Doberman (**a**), concave caudal articular surface in a clinically normal Doberman (**b**), convex caudal articular surface in a clinically normal Great Dane (**c**), sigmoid cranial articular surface in a clinically normal Doberman (**d**) and in a clinically normal Great Dane (**e**). An irregular shape of the articular surface of a CSM-affected Great Dane, classified as sigmoid, is also shown (**f**). A *white line* has been drawn to highlight the shape of the articular surfaces

In order to establish the predominant shape of the articular surfaces of the articular processes, all available images for each articular process joint from C_2–3_ to C_7_-T_1_ were reviewed. To distinguish between shapes, a straight line was drawn from the medial to the lateral edge of the articular surface. Surfaces were classified as predominantly plane (less than 0.05 cm deviation), convex or concave (deviation greater than 0.05 cm to one side of the line), or sigmoid (deviations to both sides of the line) when that particular shape was present in the majority of images (Fig. 3). This classification was adapted from an osteological study [6]. Irregular shapes were also classified as sigmoid. Minor deviations in form (approximately less than 20% of the articular surface), were discarded to reduce the influence of the edges of the articular surfaces, where the surface sometimes followed the shape of the articular process.

### Distance of the articular surfaces to the floor of the vertebral canal

As a reference to the position of the articular surfaces, the distance from the most lateral point of the articular surface of each respective articular process to the reference line was measured. While not representing the center of rotation for the joint, this point was selected as a representation of the position of the articular surface (referred to as distance) because it was the most easily identifiable edge of the articular surface for the cranial (Fig. 2, measurement a) and caudal (Fig. 2, measurement b) articular processes. The images used for these measurements were the same as the ones used to obtain angle measurements.

### Statistical analysis

Quantile-quantile plots were used to check if the variables had a normal distribution. There were no large deviations from normal and no outliers, and the dataset was therefore treated as having a normal distribution.

Means for surface angle and distance, as well as proportions of surface shapes, were compared between clinically normal Dobermans (Dob-N) and CSM-affected Dobermans (Dob-CSM), between clinically normal Great Danes (GD-N) and CSM-affected Great Danes (GD-CSM), between Dob-N and GD-N, and between Dob-CSM and GD-CSM. Comparisons were also made between functional spinal units (FSU – pair of articulating vertebrae and intervertebral disc) within each of the four groups for left and right articular processes separately. Left and right measurements were also compared per FSU for each group, using a *t*-test.

For angle and distance, these comparisons were made using multivariate ANOVA with Tukey multiple comparisons, adjusted for the height of the vertebral bodies of the dogs (for distance). For shape, chi-square tests were used to determine if there were differences between the proportions of articular surface shapes across vertebral levels between all four groups. Measurements and shape classifications were repeated, using the same MRI scans, on three randomly selected dogs from each group (*n* = 12; 20%) to calculate intraobserver agreement (after one month) using intra-class correlation and interobserver agreement using inter-class correlation. Statistical analyses were performed by a professional statistician using SAS Version 9.4. Significance was set at *P* < 0.05.

## Results

Sixty dogs were included: 30 Doberman Pinschers (15 CSM-affected and 15 clinically normal) and 30 Great Danes (15 CSM-affected and 15 clinically normal). The mean age for these dogs at the time of imaging was 6.3 years (3–12 years) for CSM-affected Dobermans; 4.3 years (2–8 years) for clinically normal Dobermans; 4 years (1–7.2 years) for CSM-affected Great Danes (mean age at onset 1.7 years); and 2.3 years (1–6.4 years) for clinically normal Great Danes.

Gender distribution for each group was: eight male and seven female CSM-affected Dobermans, eight female and seven male clinically normal Dobermans, 13 male and two female CSM-affected Great Danes, and eight male and seven female clinically normal Great Danes. Mean weight was 35 kg (26.3–50.8 kg) for CSM-affected Dobermans, 36.7 kg (26–52 kg) for clinically normal Dobermans, 57.8 kg (42–79.3 kg) for CSM-affected Great Danes, and 52.7 kg (40.5–73 kg) for clinically normal Great Danes.

The CSM-affected Dobermans had disc-associated CSM, with the main spinal cord compression at C_6–7_ (8), C_5–6_ (6), C_4–5_ (1). The main sites of spinal cord compression in the CSM-affected Great Danes, all of which had osseous-associated CSM, were C_6–7_ (8), C_4–5_ (3), C_5–6_ (2), C_2–3_ (1), and C_3–4_ (1). Seven CSM-affected Dobermans and thirteen CSM-affected Great Danes had multiple sites of compression, but the other compression sites were considered less clinically relevant.

### Angle of the articular surfaces

In general, there were no differences in the angle of the articular surfaces between Dob-N and Dob-CSM or between GD-N and GD-CSM, except for C_5–6_ left cranial and C_6–7_ except for right caudal (greater for GD-CSM). When comparing breeds, there were differences between Dob-CSM and GD-CSM at C_4–5_, C_5–6_, C_6–7_ (greater for GD-CSM), but no differences between Dob-N and GD-N (Table 1).

**Table 1:** Mean angles ± standard error (and range) of the articular surfaces per group

Level	Dogs	Left Cranial Angle	Right Cranial Angle	Left Caudal Angle	Right Caudal Angle
C_2–3_	Dob-N	26.56 ± 5.68 (5.2–37.7)	24.41 ± 7.11 (13.2–36.4)	28.35 ± 5.627 (7.6–39.2)	26.18 ± 8.156 (12.6–36.1)
	Dob-CSM	26.52 ± 5.68 (17.4–37.9)	22.68 ± 7.11 (14.9–31.5)	26.02 ± 5.62 (16.6–35)	25.54 ± 8.15 (12.9–31.9)
	GD-N	19.24 ± 5.68 (7.3–35.5)	23.18 ± 7.11 (3.5–38.3)	20.87 ± 5.62 (5.6–42.8)	23.83 ± 8.15 (0.2–41.1)
	GD-CSM	26.18 ± 5.68 (1.1–174.7)	36.43 ± 7.11 (0.3–170.7)	26.41 ± 5.62 (1.4–170.4)	46.24 ± 8.156 (2–171.9)
C_3–4_	Dob-N	24.02 ± 5.33 (11.3–45)	21.24 ± 1.91 (12.6–36.6)	23.3 ± 2.18 (8.5–45.5)	21.98 ± 1.96 (11.7–38.5)
	Dob-CSM	20.2 ± 5.33 (10.2–28.3)	21.81 ± 1.91 (13.4–31.4)	19.37 ± 2.18 (8.3–28.8)	21.81 ± 1.96 (7.3–30.2)
	GD-N	24.02 ± 5.33 (8.4–29.1)	21.24 ± 1.91 (10.3–33.7)	23.3 ± 2.18 (9.9–30.6)	21.98 ± 1.96 (11.5–35)
	GD-CSM	34.1 ± 5.33 (8.3–174.3)	21.473 ± 1.91 (0.3–35.7)	23.3 ± 2.18 (1.3–45.1)	20.97 ± 1.96 (1.1–32.5)
C_4–5_	Dob-N	23.52 ± 1.72 (10.5–37.9)	24.59 ± 1.74 (16.8–36.9)	21.50 ± 1.7 (7.5–37.9)	24.96 ± 1.75 (15.2–37.1)
	Dob-CSM	21.91 ± 1.72 ^c^ (14–30.1)	22.56 ± 1.74 ^c^ (8.5–39.7)	20.76 ± 1.7 ^c^ (11.4–30.5)	23.7 ± 1.759 ^c^ (10.6–43.9)
	GD-N	25.82 ± 1.72 (13.9–33.5)	26.69 ± 1.74 (16.7–32.3)	26.24 ± 1.7 (16.4–37.5)	26.72 ± 1.75 (21–32.8)
	GD-CSM	31.36 ± 1.72 ^c^ (19.3–42.5)	31.69 ± 1.74 ^c^ (14.5–40.7)	32.37 ± 1.7 ^c^ (22.2–43.7)	31.7 ± 1.75 ^c^ (18.4–39.3)
C_5–6_	Dob-N	27.8 ± 1.87 (18.7–41.4)	26.52 ± 2.01 (19.1–37.7)	26 ± 2.11 (18.8–39.1)	26.73 ± 2.07 (16.4–37.1)
	Dob-CSM	23.62 ± 1.87 ^c^ (12.1–41.2)	21.77 ± 2.01 ^c^ (7.7–41.9)	22.93 ± 2.11 ^c^ (11.4–41.4)	21.47 ± 2.07 ^c^ (7.4–43.2)
	GD-N	25.4 ± 1.87 ^a^ (16.4–33.3)	27.28 ± 2.01 (9.9–39.9)	24.94 ± 2.11 (12.9–33.6)	28.1 ± 2.07 (13.5–39.5)
	GD-CSM	33.58 ± 1.87 ^a, c^ (22.8–48.5)	33.04 ± 2.01 ^c^ (21.3–45.8)	31.96 ± 2.11 ^c^ (16.2–46.2)	32.07 ± 2.07 ^c^ (18.6–47.3)
C_6–7_	Dob-N	33.04 ± 2.05 (23.6–48.5)	32.42 ± 2.5 (22.1–45.4)	31.63 ± 2.43 (18.5–44.6)	31.92 ± 2.61 (21.2–43.2)
	Dob-CSM	29.36 ± 2.05 ^c^ (15.3–39.5)	30.02 ± 2.5 ^c^ (16.9–50.5)	27.4 ± 2.43 ^c^ (10.2–41.6)	28.11 ± 2.61 ^c^ (16.5–45.4)
	GD-N	29.1 ± 2.05 ^a^ (16.7–40.5)	30.45 ± 2.5 ^a^ (15.7–50.3)	27.44 ± 2.43 ^a^ (14.9–47.2)	31.56 ± 2.61 (13.4–50.8)
	GD-CSM	38.29 ± 2.05 ^a, c^ (21.8–64.4)	41.1 ± 2.5 ^a, c^ (22.9–73.4)	37.88 ± 2.43 ^a, c^ (19.5–66.5)	39.37 ± 2.61 ^c^ (18.5–78.6)
C_7_-T_1_	Dob-N	36.32 ± 2.79 (19.8–48.7)	33.24 ± 2.72 (24.5–39.8)	32.81 ± 3.04 (14.2–44.7)	30.86 ± 2.88 (17.7–39.5)
	Dob-CSM	36.92 ± 2.79 (21.4–56.3)	35 ± 2.72 (16.6–52.4)	37.84 ± 3.04 (16.9–60.3)	33.06 ± 2.88 (14.4–54.4)
	GD-N	36.15 ± 2.79 (26.5–57.4)	39.12 ± 2.72 (23.7–59.1)	33.32 ± 3.04 (18.4–51.2)	35.12 ± 2.88 (23.5–55.7)
	GD-CSM	43.83 ± 2.79 (24.6–79.8)	40.05 ± 2.72 (24.5–69.7)	41.69 ± 3.04 (21.2–79.4)	40.98 ± 2.88 (18.7–69.1)

Mean angles ± standard error adjusted for height of the vertebral bodies, with minimum and maximum values in parenthesis, for the cranial and caudal articular surfaces for clinically normal Doberman Pinschers (Dob-N) and Great Danes (GD-N) and Doberman Pinschers and Great Danes with cervical spondylomyelopathy (Dob-CSM, GD-CSM)

^a^Statistically significant difference between clinically normal and CSM-affected Great Danes (*P* < 0.05)

^b^Statistically significant difference between clinically normal Doberman Pinschers and clinically normal Great Danes (*P* < 0.05)

^c^Statistically significant difference between CSM-affected Doberman Pinschers and CSM-affected Great Danes (*P* < 0.05)

Among FSU, there was no significant difference between angles at C_6–7_ and C_7_-T_1_, which were greater than the other FSU in Dob-N. In Dob-CSM, the angle measurements were significantly greater for C_7_-T_1_ than for most other FSU, except for C_6–7_ cranial and caudal articular surfaces on the right side. Also, there were generally no significant differences in angles between FSU among Great Danes except for C_7_-T_1_ in GD-N, which was greater than the other FSU. When comparing sides, there was no significant difference in any of the groups, except for GD-N at cranial C_2–3_ and caudal C_5–6_ and C_6–7_.

Angle measurements for the cranial and caudal articular surfaces are displayed in Additional files 1, 2, 3 and 4.

### Shape of the articular surfaces

A predominant shape was recorded for all FSU except C_7_-T_1_, where the available transverse images did not consistently yield a predominant shape. There was a statistical difference in the proportion of shapes when comparing Dob-N with GD-N and Dob-CSM with GD-CSM, with an overall higher proportion of concave caudal processes in Dob-N (C_4–5_, C_5–6_, C_6–7_) compared with GD-N, and Dob-CSM (C_4–5_ and C_5–6_) compared with GD-CSM (Tables 2 and 3). Overall, there were no significant differences in the proportions of the four shapes between Dob-N and Dob-CSM or between GD-N and GD-CSM.

**Table 2:** Shape distribution for cranial articular surfaces per group

Level	Dogs	Cc (RCr)	Cv (RCr)	P (RCr)	S (RCr)	Cc (LCr)	Cv (LCr)	P (LCr)	S (LCr)
C_2–3_	Dob-N	10^b^	1	2	2^b^	10^b^	1	2	2^b^
	Dob-CSM	9^c^	2	4	0^c^	8^c^	2	5	0^c^
	GD-N	2^b^	0	2	11^b^	0^b^	0	2	13^b^
	GD-CSM	3^c^	1	1	10^c^	4^c^	3	1	7^c^
C_3–4_	Dob-N	0	14	1	0	0	13	2	0
	Dob-CSM	0	15^c^	0^c^	0^c^	0	15^c^	0	0
	GD-N	2	7	2	4	2	8	2	3
	GD-CSM	0	5^c^	5^c^	5^c^	2	8^c^	2	3
C_4–5_	Dob-N	1	12^b^	1^b^	1^b^	1	13^b^	0^b^	1^b^
	Dob-CSM	0	14	1	0	0	13	0	2
	GD-N	1	5^b^	5^b^	4^b^	0	4^b^	7^b^	4^b^
	GD-CSM	2	7	4	2	2	7	3	3
C_5–6_	Dob-N	1^b^	12	0^b^	2	1^b^	1^b^	11^b^	2^b^
	Dob-CSM	1	12	0	2^c^	0	9	0	6
	GD-N	5^b^	4	3^b^	3	2^b^	3^b^	1^b^	9^b^
	GD-CSM	2	5	2	6^c^	2	4	2	7
C_6–7_	Dob-N	0^b^	14^b^	1^b^	0	0^b^	14^b^	1^b^	0
	Dob-CSM	0	12	2	1	0	10	3	2
	GD-N	4^b^	6^b^	4^b^	1	3^b^	7^b^	4^b^	1
	GD-CSM	0	10	3	2	0	11	3	1

Shape distribution for the articular surface of the right (RCr) and left (LCr) cranial articular processes from C2-3 to C6-7 in clinically normal Doberman Pinschers (Dob-N) and Great Danes (GD-N) and Doberman Pinschers and Great Danes with cervical spondylomyelopathy (Dob-CSM, GD-CSM)

*Cc* concave, *Cv* convex, *P* plane, *S* sigmoid

^a^Statistically significant difference between clinically normal and CSM-affected Great Danes (*P* < 0.05)

^b^Statistically significant difference between clinically normal Doberman Pinschers and clinically normal Great Danes (*P* < 0.05)

^c^Statistically significant difference between CSM-affected Doberman Pinschers and CSM-affected Great Danes (*P* < 0.05)

**Table 3:** Shape distribution for the caudal articular surfaces per group

Level	Dogs	Cc (RCd)	Cv (RCd)	P (RCd)	S (RCd)	Cc (LCd)	Cv (LCd)	P (LCd)	S (LCd)
C_2–3_	Dob-N	2	6^b^	3^b^	4^b^	2	5^b^	5^b^	3^b^
	Dob-CSM	2	6^c^	4^c^	3^c^	2	3^c^	5^c^	5^c^
	GD-N	1	1^b^	1^b^	12^b^	2	0^b^	1^b^	13^b^
	GD-CSM	4	1^c^	0^c^	10^c^	5	1^c^	0^c^	9^c^
C_3–4_	Dob-N	14	0	1	0	13	0	2	0
	Dob-CSM	15	0^c^	0^c^	0^c^	15	0^c^	0	0
	GD-N	8	0	4	3	9	0	4	2
	GD-CSM	6	0^c^	4^c^	5^c^	11	1^c^	1	2
C_4–5_	Dob-N	13^b^	0	1^b^	1^b^	13	0	1	1
	Dob-CSM	15^c^	0	0^c^	0	13	0	0	2
	GD-N	5^b^	1	5^b^	4^b^	5	0	7	3
	GD-CSM	8^c^	1	5^c^	1	7	1	4	3
C_5–6_	Dob-N	12^b^	0^b^	2	1	11^b^	0	3	1^b^
	Dob-CSM	14^c^	0	0	1	12	0	0	3
	GD-N	4^b^	4^b^	3	4	4^b^	0	2	9^b^
	GD-CSM	6^c^	1	3	5	6	2	2	5
C_6–7_	Dob-N	15^b^	0	0^b^	0	15	0	0	0
	Dob-CSM	14	0	1	0	12	0	2	1
	GD-N	8^b^	2	4^b^	1	10	2	2	1
	GD-CSM	13	0	2	0	13	0	2	0

Shape distribution for the articular surface of the right (RCd) and left (LCd) caudal articular process from C2-3 to C6-7 in clinically normal Doberman Pinschers (Dob-N) and Great Danes (GD-N) and Doberman Pinschers and Great Danes with cervical spondylomyelopathy (Dob-CSM, GD-CSM)

*Cc* concave, *Cv* convex, *P* plane, *S* sigmoid

^a^Statistically significant difference between clinically normal and CSM-affected Great Danes (*P* < 0.05)

^b^Statistically significant difference between clinically normal Doberman Pinschers and clinically normal Great Danes (*P* < 0.05)

^c^ Statistically significant difference between CSM-affected Doberman Pinschers and CSM-affected Great Danes (*P* < 0.05)

In general, when comparing FSU within a group, C_3–4_, C_4–5_, C_5–6_, and C_6–7_ had a greater proportion of concave caudal and convex cranial surfaces, while C_2–3_ had comparatively more sigmoid and plane shapes. Interestingly, three CSM-affected Great Danes exhibited medially angled sigmoid caudal surfaces at C_2–3_ (*n* = 2/15) and C_3–4_ (*n* = 1/15).

### Distance of the articular surfaces to the floor of the vertebral canal

There were no statistical differences between mean distance measurements for Dob-N and Dob-CSM or between GD-N and GD-CSM. There were significant differences in mean cranial and caudal lateral distance measurements between Dob-N and GD-N and between Dob-CSM and GD-CSM, with higher values for GD-N and GD-CSM (Table 4).

**Table 4:** Mean ± standard error (and range) lateral distance of the articular surfaces per group

Level	Dogs	Left Cranial Lateral	Right Cranial Lateral	Left Caudal Lateral	Right Caudal Lateral
C_2–3_	Dob-N	1.22 ± 0.05 (1.05–1.37)	1.2 ± 0.06 (1.03–1.47)	1.32 ± 0.05 (1.09–1.46)	1.31 ± 0.06 (1.2–1.51)
	Dob-CSM	1.2 ± 0.05 (0.99–1.42)	1.15 ± 0.06 (0.83–1.44)	1.31 ± 0.05 (1.11–1.56)	1.28 ± 0.06 (1.05–1.55)
	GD-N	1.18 ± 0.05 (0.97–1.62)	1.29 ± 0.06 (0.89–1.64)	1.31 ± 0.05 (1.09–1.59)	1.41 ± 0.06 (1.01–1.79)
	GD-CSM	1.2 ± 0.05 (0.74–1.63)	1.25 ± 0.05 (0.61–1.79)	1.32 ± 0.05 (0.68–1.78)	1.34 ± 0.06 (0.56–1.93)
C_3–4_	Dob-N	0.81 ± 0.07 ^b^ (0.69–1.14)	0.81 ± 0.06 ^b^ (0.6–1.01)	0.94 ± 0.06 ^b^ (0.75–1.19)	0.95 ± 0.05 ^b^ (0.71–1.1)
	Dob-CSM	0.82 ± 0.07 (0.57–1.15)	0.86 ± 0.06 (0.66–1.07)	0.92 ± 0.06 ^c^ (0.69–1.31)	0.97 ± 0.05 ^c^ (0.76–1.17)
	GD-N	1.23 ± 0.07 ^b^ (0.81–1.52)	1.17 ± 0.06 ^b^ (0.77–1.5)	1.3 ± 0.06 ^b^ (0.94–1.62)	1.3 ± 0.06 ^b^ (0.93–1.63)
	GD-CSM	1 ± 0.07 (−0.04–1.52)	1.06 ± 0.06 (0.29–1.37)	1.24 ± 0.06 ^c^ (0.35–1.68)	1.2 ± 0.05 ^c^ (0.48–1.49)
C_4–5_	Dob-N	0.81 ± 0.05 ^b^ (0.53–1.13)	0.81 ± 0.05 ^b^ (0.62–1.08)	0.92 ± 0.05 ^b^ (0.7–1.16)	0.94 ± 0.05 ^b^ (0.76–1.19)
	Dob-CSM	0.77 ± 0.05 ^c^ (0.52–0.99)	0.73 ± 0.05 (0.5–0.95) ^c^	0.87 ± 0.05 ^c^ (0.6–1.07)	0.86 ± 0.05 ^c^ (0.61–1.06)
	GD-N	1.19 ± 0.05 ^b^ (0.85–1.44)	1.18 ± 0.05 ^b^ (0.83–1.49)	1.32 ± 0.05 ^b^ (1.07–1.71)	1.31 ± 0.05 ^b^ (1.06–1.62)
	GD-CSM	1.33 ± 0.05 ^c^ (0.81–1.81)	1.3 ± 0.05 ^c^ (0.7–1.72)	1.47 ± 0.05 ^c^ (0.98–2.09)	1.41 ± 0.05 ^c^ (0.89–1.89)
C_5–6_	Dob-N	0.83 ± 0.06 ^b^ (0.43–1.26)	0.83 ± 0.06 ^b^ (0.55–1.14)	0.94 ± 0.05 ^b^ (0.59–1.31)	0.93 ± 0.06 ^b^ (0.7–1.18)
	Dob-CSM	0.71 ± 0.06 ^c^ (0.36–0.99)	0.69 ± 0.06 ^c^ (0.39–0.98)	0.82 ± 0.05 ^c^ (0.5–1.11)	0.81 ± 0.06 ^c^ (0.52–1.05)
	GD-N	1.3 ± 0.06 ^b^ (0.88–1.52)	1.34 ± 0.06 ^b^ (−0.51–0.22)	1.44 ± 0.06 ^b^ (1.06–1.68)	1.5 ± 0.06 ^b^ (1.03–2.11)
	GD-CSM	1.5 ± 0.06 ^c^ (0.92–1.99)	1.5 ± 0.06 ^c^ (1.02–1.86)	1.6 ± 0.06 ^c^ (1.02–2.09)	1.6 ± 0.06 ^c^ (1.11–1.97)
C_6–7_	Dob-N	0.78 ± 0.04 ^b^ (0.55–0.91)	0.78 ± 0.05 ^b^ (0.6–1.03)	0.89 ± 0.04 ^b^ (0.69–1)	0.89 ± 0.05 ^b^ (0.7–1.12)
	Dob-CSM	0.79 ± 0.04 ^c^ (0.42–1.18)	0.82 ± 0.05 ^c^ (0.44–1.22)	0.91 ± 0.04 ^c^ (0.5–1.18)	0.92 ± 0.05 ^c^ (0.5–1.28)
	GD-N	1.18 ± 0.04 ^b^ (0.69–1.44)	1.23 ± 0.05 ^b^ (0.83–1.67)	1.34 ± 0.04 ^b^ (0.9–1.49)	1.38 ± 0.05 ^b^ (0.99–1.78)
	GD-CSM	1.36 ± 0.04 ^c^ (0.99–1.59)	1.38 ± 0.05 ^c^ (0.92–1.67)	1.47 ± 0.04 ^c^ (1.05–1.68)	1.53 ± 0.05 ^c^ (1.07–1.88)
C_7_-T_1_	Dob-N	1.02 ± 0.06 ^b^ (0.65–1.33)	1.05 ± 0.05 ^b^ (0.74–1.35)	1.1 ± 0.06 ^b^ (0.76–1.44)	1.15 ± 0.05 ^b^ (0.81–1.46)
	Dob-CSM	1.12 ± 0.06 ^c^ (0.88–1.39)	1.11 ± 0.05 ^c^ (0.9–1.43)	1.23 ± 0.06 ^c^ (0.96–1.45)	1.2 ± 0.05 ^c^ (0.98–1.56)
	GD-N	1.45 ± 0.06 ^b^ (1.26–1.81)	1.51 ± 0.05 ^b^ (1.27–1.84)	1.54 ± 0.06 ^b^ (1.36–1.89)	1.61 ± 0.05 ^b^ (1.42–1.92)
	GD-CSM	1.55 ± 0.06 ^c^ (1.1–2.12)	1.58 ± 0.05 ^c^ (1.07–2.12)	1.67 ± 0.06 ^c^ (1.22–2.21)	1.7 ± 0.05 ^c^ (1.27–2.19)

Mean ± standard error adjusted for height of the vertebral bodies, with minimum and maximum values in parenthesis for lateral distance of the articular surface of cranial and caudal articular processes, given as the distance to the floor of the vertebral canal (reference line), in clinically normal Doberman Pinschers (Dob-N) and Great Danes (GD-N) and Doberman Pinschers and Great Danes with cervical spondylomyelopathy (Dob-CSM, GD-CSM), in centimeters

^a^Statistically significant difference between clinically normal and CSM-affected Great Danes (*P* < 0.05)

^b^Statistically significant difference between clinically normal Doberman Pinschers and clinically normal Great Danes (*P* < 0.05)

^c^Statistically significant difference between CSM-affected Doberman Pinschers and CSM-affected Great Danes (*P* < 0.05)

Among FSU, both groups of Dobermans usually had higher values for C_2–3_, together with or followed by C_7_-T_1_, and then C_3–4_ through C_6–7_, with no difference from C_3–4_ through C_6–7_ for cranial and caudal lateral distances. In GD-N, measurements at C_7_-T_1_ were generally higher, followed by (cranial) or equal to (caudal) those for C_2–3_. In GD-CSM, C_7_-T_1_ values were higher for cranial and caudal lateral reference points, followed by C_2–3_. Comparison between sides showed no significant difference overall.

Distance measurements for the cranial and caudal articular surfaces are available in Additional files 5, 6, 7 and 8.

### Intraobserver and interobserver agreement

Intra-class correlations for measurements were generally greater than 0.95 for intraobserver agreement. The highest correlation was seen for the lateral distance for the left cranial articular process (0.9765) and the lowest for the right cranial articular surface angle (0.9090). Shape identifications were 80% consistent, with the least consistency observed for the cranial left articular surfaces (73%) and the highest for the caudal left (85%).

For interobserver agreement, inter-class correlations for measurements were 0.8 in average, with the highest correlation for the lateral distance of the left cranial articular process (0.9373) and the lowest for the right caudal articular surface angle (0.6). Interobserver shape identifications were 69% consistent, with the least consistency observed for the caudal right (62%) and the highest for the cranial left articular surface (75%).

## Discussion

In this study, we investigated the angles, position (as given by lateral distance) and shapes of the articular surfaces of the cervical vertebral articular processes in Dobermans and Great Danes with and without CSM. Such a thorough investigation of these characteristics and their relationships with CSM had not been previously performed.

Although our second hypothesis, that there would be a difference between breeds, was confirmed, our primary hypothesis, that there would be significant differences in articular surface shape, angle, and position between CSM and non-CSM-affected dogs, was not confirmed.

Overall, there were no significant differences in angle, lateral distance, or predominant shape of the articular surfaces between Dob-N and Dob-CSM or between GD-N and GD-CSM, suggesting that biomechanical differences between dogs with and without CSM are likely not related to these characteristics in these breeds. These findings also suggest that a difference in the overall size of the articular processes as a consequence of the osseous changes commonly seen in CSM-affected Great Danes [10, 13], may not result in a significant difference in position of the articular surface itself.

Overall, the shape of the articular surfaces was not associated with clinical disease in either breed. There was, however, a significant difference in shape patterns between breeds (comparing Dob-N to GD-N and Dob-CSM to GD-CSM). In general, Dobermans had a greater proportion of concave caudal surfaces, which has been associated with a greater capacity for axial rotation [6, 14]. The higher proportion of concave caudal surfaces may explain why Dobermans typically have disc-associated CSM, whereas this form of CSM is uncommon in Great Danes. The shape of the articular surfaces may be partially responsible for the higher torsional forces seen in the caudal cervical region [15], facilitating development of intervertebral disc degeneration and protrusion in this region.

Interestingly, we found three medially angled sigmoid caudal surfaces in GD-CSM. These were considered the result of osseous proliferation with severe deformation of the articular process, although it cannot be ruled out that they could be an anatomical variant. However, this would likely not be related to the change in orientation observed in humans [4, 5] which does not occur in dogs [6, 16]. Also, sigmoid surfaces were observed as being medially concave-laterally convex or medially convex-laterally concave, with both sometimes present in the same dog, which had not been previously described [6].

Limitations of this study include a limited number of dogs per group. A greater number of subjects might have resulted in additional significant differences between clinically normal and CSM-affected dogs. Nonetheless, these numbers are consistent with previous comparative studies focusing on CSM [8, 17, 18]. A power analysis after completion of the study obtained a power of 75%. This power was considered adequate for this specific study.

The use of a single observer, though reducing variability, can be considered another limitation; however, intraobserver agreement indicated a high degree of reliability in the measurements and interobserver agreement was considered good for the majority of the measurements [19]. Although another limitation can be the selection of a single image of the articular surface for the measurements, which would not be representative of the variations within a joint, this permits standardization of the measurement method and is consistent with other studies that looked at articular surface angles [11, 12].

Other comparisons, such as between sites with and without spinal cord compression or between the sites immediately cranial or caudal to a site of spinal cord compression and sites that were not adjacent to a site of spinal cord compression, would have been interesting, however, there were not enough compression sites in CSM-affected Dobermans or sites without compression in CSM-affected Great Danes to make such comparisons worthwhile. Furthermore, joining all sites of compression regardless of location (C_2–3_, C_3–4_, C_4–5_, C_5–6_, C_6–7_, C_7_-T_1_) would likely result in another type of error since most of the compression sites were in the caudal cervical vertebrae.

## Conclusions

Differences in angle, shape, and position of the articular surfaces between Dobermans and Great Danes suggest that the movements of the caudal cervical region of the vertebral column may differ between these breeds. The higher proportion of concave articular surfaces in Dobermans may explain why this breed has a higher proportion of disc-associated CSM compared to Great Danes.

Considering that no differences in angle, shape or position of the articular processes were found between normal and CSM-affected Dobermans or Great Danes, their relevance to the development of CSM in these breeds appears to have a secondary role.

## Additional files

Additional file 1: Angle of the cranial and caudal articular surfaces to the reference line for clinically normal Doberman Pinschers. Angle measurements for the articular surface of the right (R) and left (L) cranial and caudal articular processes in 15 clinically normal Doberman Pinschers (Dob-N). (XLSX 18 kb)

Additional file 2: Angle of the cranial and caudal articular surfaces to the reference line for Doberman Pinschers with cervical spondylomyelopathy. Angle measurements for the articular surface of the right (R) and left (L) cranial and caudal articular processes in 15 Doberman Pinschers with cervical spondylomyelopathy (Dob-CSM). (XLSX 18 kb)

Additional file 3: Angle of the cranial and caudal articular surfaces to the reference line for clinically normal Great Danes. Angle measurements for the articular surface of the right (R) and left (L) cranial and caudal articular processes in 15 clinically normal Great Danes (GD-N). (XLSX 18 kb)

Additional file 4: Angle of the cranial and caudal articular surfaces to the reference line for Great Danes with cervical spondylomyelopathy. Angle measurements for the articular surface of the right (R) and left (L) cranial and caudal articular processes in 15 Great Danes with cervical spondylomyelopathy (GD-CSM). (XLSX 18 kb)

Additional file 5: Distance from the lateral edge of the cranial and caudal articular surfaces to the reference line for clinically normal Doberman Pinschers. Distance (in centimeters) from the lateral edge of the articular surface of the right and left cranial and caudal articular processes to the reference line in 15 clinically normal Doberman Pinschers (Dob-N). (XLSX 23 kb)

Additional file 6: Distance from the lateral edge of the cranial and caudal articular surfaces to the reference line in Doberman Pinschers with cervical spondylomyelopathy. Distance (in centimeters) from the lateral edge of the articular surface of the right and left cranial and caudal articular processes to the reference line in 15 Doberman Pinschers with cervical spondylomyelopathy (Dob-CSM). (XLSX 24 kb)

Additional file 7: Distance from the lateral edge of the cranial and caudal articular surfaces to the reference line in clinically normal Great Danes. Distance (in centimeters) from the lateral edge of the articular surface of the right and left cranial and caudal articular processes to the reference line in 15 clinically normal Great Danes (GD-N). (XLSX 23 kb)

Additional file 8: Distance from the lateral edge of the cranial and caudal articular surfaces to the reference line in Great Danes with cervical spondylomyelopathy. Distance (in centimeters) from the lateral edge of the articular surface of the right and left cranial and caudal articular processes to the reference line in 15 Great Danes with cervical spondylomyelopathy (GD-CSM). (XLSX 23 kb)

## Abbreviations

CSM: Cervical spondylomyelopathy
DA-CSM: Disc-associated cervical spondylomyelopathy
Dob-CSM: Cervical spondylomyelopathy-affected Doberman Pinschers
Dob-N: Clinically normal Doberman Pinschers
FSU: Functional spinal unit
GD-CSM: Cervical spondylomyelopathy-affected Great Danes
GD-N: Clinically normal Great Danes
MR: Magnetic resonance
MRI: Magnetic resonance imaging
OA-CSM: Osseous-associated cervical spondylomyelopathy
